# Recent Advances in Intelligent Source Code Generation: A Survey on Natural Language Based Studies

**DOI:** 10.3390/e23091174

**Published:** 2021-09-07

**Authors:** Chen Yang, Yan Liu, Changqing Yin

**Affiliations:** School of Software Engineering, Tongji University, Shanghai 201804, China; yangchensa@tongji.edu.cn (C.Y.); yinchangqing@tongji.edu.cn (C.Y.)

**Keywords:** natural language-based source code generation, systematic literature review, machine learning application

## Abstract

Source Code Generation (SCG) is a prevalent research field in the automation software engineering sector that maps specific descriptions to various sorts of executable code. Along with the numerous intensive studies, diverse SCG types that integrate different scenarios and contexts continue to emerge. As the ultimate purpose of SCG, Natural Language-based Source Code Generation (NLSCG) is growing into an attractive and challenging field, as the expressibility and extremely high abstraction of the input end. The booming large-scale dataset generated by open-source code repositories and Q&A resources, the innovation of machine learning algorithms, and the development of computing capacity make the NLSCG field promising and give more opportunities to the model implementation and perfection. Besides, we observed an increasing interest stream of NLSCG relevant studies recently, presenting quite various technical schools. However, many studies are bound to specific datasets with customization issues, producing occasional successful solutions with tentative technical methods. There is no systematic study to explore and promote the further development of this field. We carried out a systematic literature survey and tool research to find potential improvement directions. First, we position the role of NLSCG among various SCG genres, and specify the generation context empirically via software development domain knowledge and programming experiences; second, we explore the selected studies collected by a thoughtfully designed snowballing process, clarify the NLSCG field and understand the NLSCG problem, which lays a foundation for our subsequent investigation. Third, we model the research problems from technical focus and adaptive challenges, and elaborate insights gained from the NLSCG research backlog. Finally, we summarize the latest technology landscape over the transformation model and depict the critical tactics used in the essential components and their correlations. This research addresses the challenges of bridging the gap between natural language processing and source code analytics, outlines different dimensions of NLSCG research concerns and technical utilities, and shows a bounded technical context of NLSCG to facilitate more future studies in this promising area.

## 1. Introduction

As the cost of data ingestion, storage and computation continues to decrease, applying AI in practice is becoming the focus of the whole IT industry. The enormous potential is unleashed with a combination of AI and other fields, and Source Code Generation (SCG) is no exception. Various deep learning algorithms and neural network construction approaches have been applied to the automatic SCG and intelligent software development environment. SCG allows practitioners to generate source code from a higher level of abstraction [1]. Processing methods more intelligent thatn SCG are opening new possibilities for the input abstraction levels, resulting in the endless emergence of new perspectives. Popular perspectives represented by specific descriptions can be instantiated as domain-specific language, formal language, modeling language, natural requirements, etc. Among these, natural requirements, including natural language or prototypes, have attracted intense academic interest due to their intuitiveness, roughness, and primitiveness.

**Figure 1:**
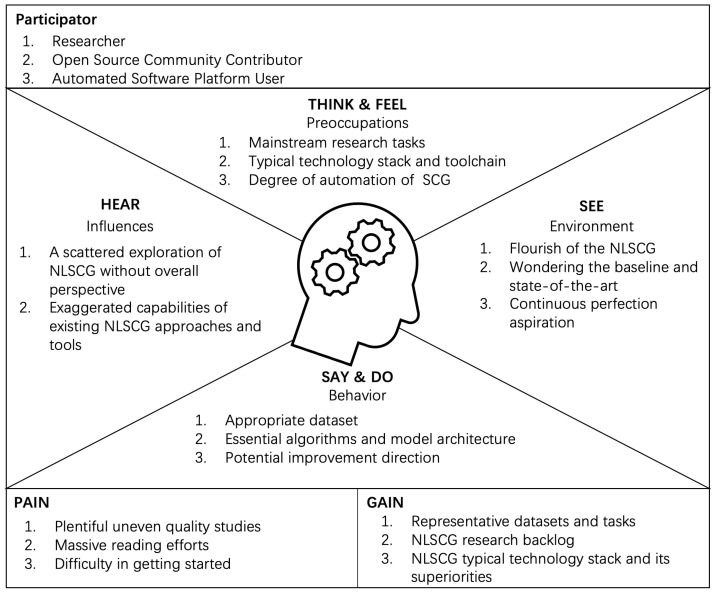
NLSCG empathy map.

As the ideal scenario for automatic SCG, research on Natural Language-based Source Code Generation (NLSCG) is booming. Some encouraging progress has been made, developing promising solutions with a confident performance. The majority of these studies start with problems of individuation coupled with specific datasets, presenting the tendency of tentative occasional successful solutions. There is no systematic study to promote the further development of this field. To understand the practitioners interested in NLSCG from their perspective, we brainstormed and sketched out an empathy map (see Figure 1) to direct the following research concerns and trends. As the empathy map expressed, participators, including researchers for typical NLSCG models, open-source community contributors for NLSCG products and toolchains, and automated software platform users for considering the degree of automation, are passionate about the NLSCG field. Under the mainstream trend of vigorous development of NLSCG, participants are curious about the baseline and the state-of-the-art NLSCG models.

However, the abundant relevant studies and tools driving the process make it more laborious and challenging for participants to obtain comprehensive and detailed NLSCG research actualities. They confront bad quality studies and tools, and have to devote massive amounts of effort to reading; hence, there are difficulties in quickly getting started. Trying to discern the outstanding direction of improvement from partial studies calls to mind the story of the blind men and the elephant. The improvements may inadvertently fall into a futile pitfall, so it is meaningless to promote the entire field. It is urgent to clarify the current development status of NLSCG, namely, the appropriate dataset, the essential algorithms and representative architecture of the transformation model, the living bottlenecks, the enabling factors, and the potential perfection directions. Based on these motivations, we investigate the actualities and future trends, summarize representative datasets and tasks, and gain insights from the research backlog of NLSCG. Finally , the NLSCG landscape depicts typical components, their correlations, and their advantages.

NLSCG allows users to generate source code from an extremely intuitive and primitive perspective. This end-to end manner is amicable for those who lack prior knowledge in source code. NLSCG processing techniques can also be applied to commercial automated software platforms, enabling us to establish natural language interfaces with executable source code, which can reduce the cost of learning and the training cost to a certain extend. Research in this field shows its significance, as the learned experiences would be applied to similar scenarios that transform from abstract and fuzzy descriptions to highly structural constrained representations. In addition, widespread application of deep learning has promoted the prosperity of the transformation between natural language and source code like semantic code search [2] and automatic comment generation [3]. However, natural language precisely challenges the efficient construction of transformation models due to its ambiguity and uncertainty. The well formedness, type-sensitiveness, executability [4], and semantics of source code create the most difficulties. Many studies [4,5,6,7,8,9,10,11,12,13,14,15,16,17,18,19,20,21,22,23,24,25,26,27,28,29,30,31,32,33,34,35,36,37,38,39,40,41,42,43,44,45,46,47,48,49,50,51,52,53,54,55,56,57,58,59,60,61,62,63,64,65,66,67,68,69] have been conducted on the NLSCG and its relevant tasks by employing baseline and state-of-the-art deep learning models and algorithms. Abundant relevant studies are creating momentum to make this the right moment to explore the entire NLSCG field, with the expectation of discovering its actualities, potentials, and improvements.

This paper makes the following contributions: (1) We position the role of NLSCG and specify the context among various SCG genres empirically based on the domain knowledge and programming considerations. (2) We clarify the NLSCG field via an elaborate literature survey and get a distinct problem understanding. (3) We model the NLSCG research problems by explicating technical focus and adaptive challenges and gaining insights from the research backlog. (4) We summarize the technology landscape of NLSCG through exhaustive research on the transformation model and depict the critical tactics used in the essential components and their correlations.

The remainder of this paper is organized as follows. We introduce our research journey which guides our subsequent works in Section 2. In Section 3, various code generation genres are introduced before we derive a definition of NLSCG. Section 4 strengthens the process of NLSCG problem understanding through a systematic literature tracking approach. We elaborate on the insights from the research backlog in Section 5. In Section 6, the state-of-art NLSCG technology landscape and essential components along the generating pipeline are depicted, and this survey is concluded in Section 7.

## 2. Research Journey and Context

**Figure 2:**
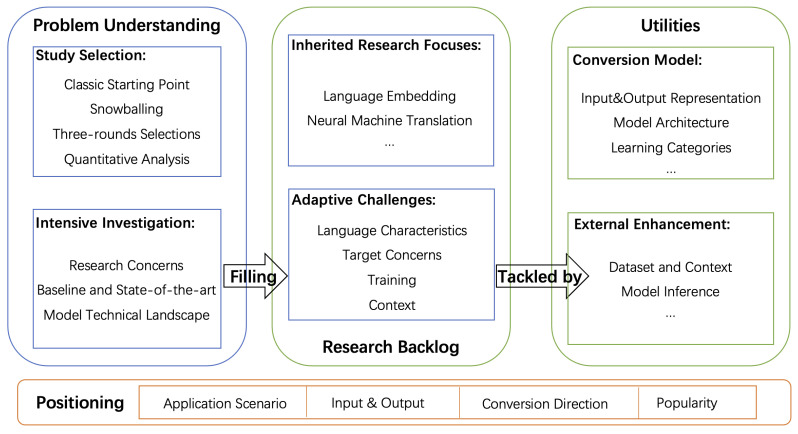
The Research Journey for this Survey.

Figure 2 presents the research journey we followed in this survey. The journey starts with explaining the various SCG genres to clearly examine the research vision, in which we position the role of NLSCG among these genres. To figure out the state-of-the-art and potentials of NLSCG exhaustively, we launched an exploration via three phases: (1) Problem understanding; (2) research backlog; and (3) utilities. The three phases are present in succession; the conclusion obtained by the former is considered as the basis and constraints for the latter, while the latter echoes the former.

Problem Positioning: Various SCG genres constantly emerge and develop, contributing to the building of the software engineering automation ecosystem. Gazing at the ecosystem from the overall perspective, we specify the NLSCG context, expanding the entire research vision, which implicates specific impacts on NLSCG research concerns and technical means. Based on the understanding and analysis of automation software engineering, we position the role of NLSCG among the various SCG genres in terms of the application scenarios, the input and output style, the conversion direction, and the popularity, and finally propose a preliminary definition of NLSCG.

Problem Understanding: We leverage the snowballing literature collection method and its matched analysis to enhance the problem understanding. NLSCG possesses a small domain scope and presents a certain homogeneity technical tendency. The very classic and well-known studies in the NLSCG field are selected before the snowballing process is executed . Afterward, depending on the predefined selection rules (expected to be three-round selections), the literature snowballing continuously until the appropriate significant studies are screened out. Next, we perform quantitative analysis to get critical statistical information from these potential studies. After completing these peripheral tasks, we investigate these selected studies intensively with specific purposes, laying a foundation for our subsequent works. These purposes include exploring research concerns, technical landscape, baseline, and state-of-the-art model details. We abstract the crude prototype of NLSCG backlog items from the research concerns and summarize the NLSCG utilities from model-relevant attempts. We build up a deepening understanding of the NLSCG research context from these investigations, and it paves the way for better exploration and summarization of our subsequent steps.

Research Backlog: To identify the ongoing concerns of the NLSCG field and consequently understand current efforts and potential directions of future improvement, we present the research backlog for a more vivid expression. Adapting the term “backlog” coined from agile development, we consider it the “research to-do task” in this NLSCG field. Through series of readings on the selected studies collected by the snowballing process, we accumulate the research backlog in terms of resource, environment, boundary, enabler, utilities, etc., with the research concerns as a clue. We tentatively extract a taxonomy based on scattered research backlog items from the perspectives of inherited research focuses and adaptive challenges. While this “taxonomy” exhibits immaturity and fragility, it can be regarded as a feasible scheme to collect the research backlog items. Finally, we summarize insights gained from the NLSCG research backlog and elaborate the details in conjunction with specific studies. The research backlog deepens the understanding of the NLSCG problem and contributes to sketching up the essential utilities for NLSCG.

Utilities: As the critical part of the NLSCG solutions, the design and technical implementation details of the transformation models have shown steady attraction for relevant practitioners. Several aspects are particularly remarkable, including the baseline and essential components indicating how the model sprouts; how the advanced models are explored from different angles; how the fuzzy mappings between research concerns and implementations function; and the tactics adopted by every pivotal component. Similarly, we gain much raw information about NLSCG model attempts based on the selected studies from the snowballing. Taking the research backlog as a guideline, we decompose the raw information, convert it into essential components, and then build interactions according to the data processing chain and the conventional deep learning model pipeline. Finally, we summarize and depict all critical tactics and give a detailed explanation of the transformation model, and we regard them as the technical utilities of NLSCG. The utilities can tackle the current backlog items from two directions: Internal model advancement and external enhancement. Apparently, advancements deriving from the transformation model, such as input and output representation, model architecture, learning categories, etc., are worth great effort. Besides, embracing diverse and divergent thinking upon these predefined directions until covering all mainstream processing manners is also an indispensable part of investigating the essential utilities. Some studies go in the opposite direction, searching for probable routes of external enhancement, such as the dataset quality that determines the upper-bounds of the model performance, the retrieval strategy in the model inference stage, etc. The essential utilities would bring a comprehensive understanding of NLSCG models to beginners, and, what is more, it would inspire practitioners with more potential improvements in this field.

## 3. Source Code Generation Genres

**Figure 3:**
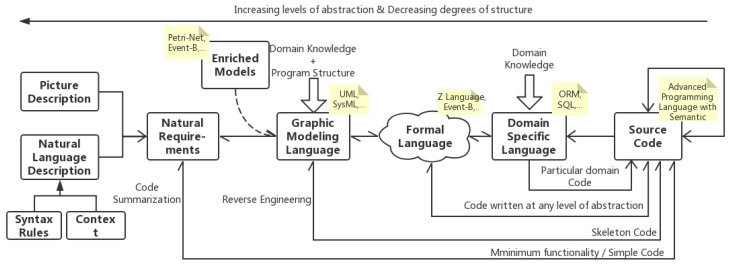
Source Code Generation Genres.

Various description systems are proposed to depict, design, and refine software objects and artifacts, and these description systems are then considered as the input end of SCG. Automatic generating source code from various description styles is a historical field both in academic and industrial areas. Limited by the characteristics of the description system, different approaches present various tendencies. According to automation software engineering status, the dominating SCG genres and their correlations are shown in Figure 3. Overall, the abstract level of description increases from the direction of right-to-left in the pipeline. The farther the horizontal distance between the two description systems in Figure 3, the more difficult it is to convert between them due to their vast degree of abstraction. Different SCG genres show their unique application scenario and popularity and are coupled with the technical conditions at the time. Some genres are still active, like NRA, DSLA, and GMA, while others are gradually lacking attention like FLA and EMA. Besides, the transformations between different description systems also attract a certain amount of attention, such as code summarization, reverse engineering, etc. We summarized the primary SCG genres as follows.

Domain-Specific Language based Approach (DSLA): As the most commonly applied approach in the industry, the transformation between domain-specific language (DSL) and source code tends to be business friendly and to involve close-domain couplings. DSL specifies a set of domain-related rules that the software must obey and run on a dedicated, high-reliability scenario [70], presenting extreme precision of transformation. This would achieve more cost effectiveness during the development process. From another perspective, the transformation is driven by rules manually defined by domain experts and would bring a higher cost of learning in the initial stage.

Formal Language based Approach (FLA): The formal method describes and analyzes the behavior of software systems utilizing mathematical symbols, and the verifiability is capable of improving the correctness of the system [71]. FLA possesses high accuracy under specific domains and fits relatively common scenarios. However, formal language-based methods and techniques raise a high demand for practitioners, resulting in abundant learning and training efforts to adapt to the rigid limitation. They have to make trade-offs between the production cost and effectiveness, and this limitation narrows the scope of application in the industry area.

Graphic Modeling-based Approach (GMA): Generating source code from modeling languages supporting object-oriented analysis and design is a classical direction. This approach focuses on the high-level design of systems, for instance, the static skeleton code (like class declaration, function definition, etc.) of a specific scenario. Relatively complex research can produce the business process as well [72]. The main challenge in GMA is to replenish the details of the generated code [73]. GMA possesses a more substantial universality independent of implementation details and could adapt to various software development scenarios. However, conversions between the two highly abstract description systems will lead to unavoidable information loss. In addition, GMA pays close attention to static mapping, and the generated source code with the simple logic flow cannot recreate the details, making it infeasible to be applied in practical application.

Enriched Models based Approach (EMA): It has been a long time since UML has been applied to design object-oriented systems. However, the ambiguity of models created by UML specifications makes it inappropriate for it to be the starting point of automatic code generation [74]. Models attached by strictly defined mathematical concepts and objects, such as Petri net, are promising directions for improvement. EMA focuses on the rigorous mapping of UML notations to the object-oriented class of enhanced models. While limited by the restricted structure of models and exponential efforts while constructing the reachability graphs, EMA can still greatly enhance the accuracy, readability, or extensibility of the generated code in some instances.

Nature Requirement based Approach (NRA): As continues on semantic parsing and deep learning, NRA has become one of the most active research topics in SCG. The natural requirement can be divided into prototype figure description [75] and natural language description. It is the most intuitive approach to generating source code from natural language, especially for programming beginners. NRA departs from past work on methodology and mode of thinking for the primitive, completely undesigned input end. At present, NRA tends to generate simple code snippets which implement minimum functionalities with unsteady performance. Researchers are trying to improve the transformation performance by attaching syntactic constraints and contextual information.

In the SCG genre diagram, various description forms and their conversion paths are highlighted. We describe the conversion direction, pros, and cons of each SCG genre in terms of the description systems features and research actualities. Each genre has its characteristics and has enlivened recent studies in certain areas, but they present as more outmoded than NRA. NRA, especially the natural language description-based approach, is the most popular, promising, and worthy of consideration. The challenges confronted by natural language-based source code generation (NLSCG) is foreseeable; we tentatively propose the definition of NLSCG as follows:

Given a specific problem context C (C can not be specified in the code snippet generation and program synthesis task types, refer to Section 4.3) and a natural language description NL to C, natural language-based source code generation (NLSCG) converts the input NL into the output executable source code (SC) corresponding to that NL. During the generation process, the semantic integrity of NL, the well-formedness, type-sensitiveness, executability of SC, and the semantic consistency between the two ends must be guaranteed. In addition, when C is given, NL must embed appropriate information from C, and the compatibility of SC and C must be guaranteed.

## 4. Problem Understanding Process

Abiding by the research journey map, the process started with well-designed research snowballing with the goal of acquiring valuable studies. We carried out the iterative forward and backward snowballing to obtain a mass of studies and employed three round screening to get small minority studies suitable for follow-up research. These selected studies are subsequently utilized for quantitative analysis to obtain preliminary viewpoints on recent trends in the NLSCG field. More importantly, these preliminary investigations have contributed to the refinement of NLSCG task types based on the basic NLSCG definition. There are slight differences between these types in research concerns and modeling priorities. Finally, we payed attention to the indispensable dataset for constructing the NLSCG models and summarized 36 datasets as well as their foundational information and applied task types.

### 4.1. Snowballing Process

Recently, an increasing number of studies have been published in the open-source database. To collect studies related to intelligent code generation and its similar tasks scientifically and effectively, we utilize the snowballing approach [76]. A very classic and well-known paper LPN [5] is selected as the starting point of the snowballing process. LPN proposes a novel NLSCG neural architecture considering the conditioning context and granularity of generation, and the creation of two new datasets, especially Hearthstone, has launched a new era for the NLSCG field.

**Figure 4:**
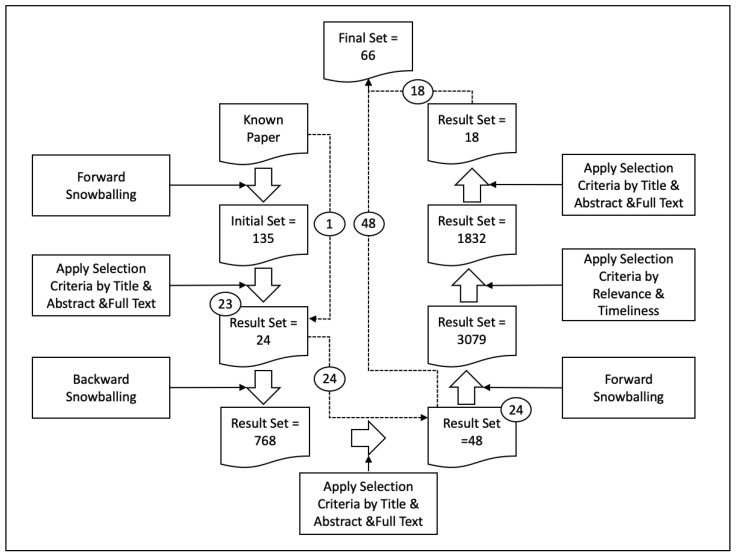
NLSCG Snowballing Process (until 31 May 2020).

The whole literature snowballing process and selection result sets are shown in Figure 4. With the starting point of LPN, by utilizing forward snowballing, we collected 135 citations. In the next round of screening, 24 studies were retained after applying selection criteria by title, abstract, and full text by turns. Then we conducted backward snowballing. We collected 768 references and retained 24 studies after removing duplicates and screening by title, abstract, and full text by turns. After this round of screening, 48 studies were retained in total. To prevent possible omissions and obtain the latest results, we carried out a new round of forward snowballing and collected 3079 citations. Afterward, we conducted a round of screening in advance by Google Scholar relevance and timeliness for the sake of effectively filtering. Finally, through a full round of three-level screening, 66 studies were selected (the complete list of the selected studies is available online: Selected Studies List Ref: https://github.com/Ada12/NLSCG_Research/blob/main/selected-studies-list.md, accessed on 20 August 2021 ).

### 4.2. Studies Analysis

**Figure 5:**
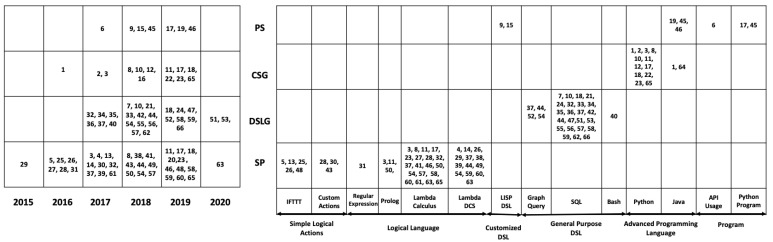
Selected Studies Distribution between Publication Year, Generation Task and Dataset.

This section conducted a simple quantitative analysis of the selected studies from publication year, adopted datasets, and generation task dimensions. We summarized the development trend of automatic code generation in recent years and the most classic dataset classification through an intuitive two-dimensional diagram. As shown in Figure 5, the serial number in each grid represents the selected research ID, and the left half of the horizontal axis represents the publication year. The dataset type present on the right and the vertical axis represents the type of generation task described in Section 4.3. Besides, we summed up the utilized datasets by different studies to provide references for future readers. Section 4.4 elaborates the detail of all datasets used in selected studies.

Studies are classified into at least one code generation task type according to the adopted datasets and their introductions. The left half of Figure 5 shows the study IDs related to the four code generation task types in the past five years. Only one study was published in 2015, and since then the number of published studies has been increasing continuously, especially in the years 2018 and 2019. Due to the screening time range limitation, only a small part of the 2020 studies was selected. Some studies tend to research domain-general problems across different tasks, using multiple types of datasets. The most conspicuous representatives are studies 17 [6] and 18 [7], which employ various datasets across three task types.

The right half of Figure 5 shows the study IDs with specific generation task types and the target datasets. The output language corresponding to the dataset becomes increasingly complicated from the left-to-right direction. The complexity of syntax, the richness of semantic, the length of utterance-denotation pairs, and more importantly, the locality design pattern and the global architecture tactics make different ensemble generation languages with a qualitative leap. In general, to achieve the goal of code generation tasks, various datasets are proposed as the basis for training in this area. The lambda calculus and its variants lambda DCS, SQL, and Python are the most popular generation style. It is worth noting that program synthesis takes customized DSL [8,9] as a springboard for better bridging the huge abstraction degree gap between natural language and programs.

### 4.3. Natural Language-Based Intelligent Code Generation Tasks

Considering SCG tasks as machine translation [77] has kicked off a generation storm tide by introducing deep learning to this field. Deep learning has gradually become the most commonly used technology for such tasks, and impressive performance has been achieved. However, various output end styles tend to adopt different problem-solving tactics and impact the design of neural network models. Therefore, in the field of intelligent NLSCG and relevant tasks, we summarize four mainstream types from selected studies in terms of output end.

Semantic Parsing (SP): Semantic parsing is a classical school of computational linguistics and has always been an active research area. It maps natural language utterances into a semantic representation [76]. In the last few years, researchers have been trying hard to generate semantic representations such as regular expressions, if–then recipes, lambda calculus with their semantics, etc. The expressions generated by semantic parsing are associated with simple logic generally. The fuzzy mapping between each utterance and its logical form makes it more challenging. The relative entropy symmetry between the expressions and natural language gives more opportunities to produce a perfect semantic parser. However, researchers are encountering difficulties when dealing with complicated symbolic expressions [10].

Domain Specific Language Generation (DSLG): Generating types under the constraints of inherent modes usually take DSL as a medium. Currently, SQL generation is the most accessible direction, and this type of generation is limited to some meaningful fields under the DSL framework. On the other hand, DSL generation can be considered as the sub-stage of the program synthesis process. By customizing the DSL suitable for the current generation target and context, the stage of DSL generation can be regarded as a buffer from ambiguous natural language to the high-level patterns in the program. DSLG is capable of assisting in program synthesis by bridging the gap.

Code Snippet Generation (CSG): Code snippet generation focuses on producing a small part of programs, which refers to fragments without advanced business logic flow in a project-level perspective. Researchers have tried to generate general-purpose languages like Python and Java, and are also starting to deliver impressive results. Compared with semantic parsing, the generation of this kind of code (1) usually involves a relatively large code scale and long dependency chain; and (2) encounters more complex generation scenarios. What is more, source code snippets are tightly constrained by syntax rules and affected by context. Moreover, numerous expressions and statements increase the difficulty of generation.

Program Synthesis (PS): Program generation is the ultimate goal and the thorny branch in the field of intelligent code generation. The asymmetries of information entropy between the program and natural language aggravate the difficulties of program synthesis. The mixture of the high-level patterns in the program and low-level detail implementations makes it extremely challenging to generate a one-shot result based on natural language. Therefore, a two-stage method was proposed to solve such problems [7,11].

### 4.4. Source Code Generation Relevant Datasets

There are multiple existing cross-domain datasets coupling with intelligent code generation tasks. To inspire in-depth research about this direction in readers, we combed all datasets appearing in selected studies. We created a profiling table for all selected studies, and purposely extracted and gathered information filled into the corresponding grid. We collected 21 attributes of different granularities from each study, including the adopted datasets. By integrating and removing the duplicate datasets, finally we obtain all the adopted datasets (36 in total). Due to the differences between the target language’s complexity, a wide variation exists in intelligent generation model design, including the algorithm, the intermediate representation, and critical processing procedures. Therefore, these datasets and their variants are applied to different kinds of SCG tasks. Based on the characteristics and application fields of the datasets, we launched a finer-grained classification. Many classic datasets among them have been applied in semantic parsing tasks for years. Meanwhile, there are also emerging datasets created for current intelligent NLSCG. Aiming at the code generation task and according to the output end language complexity, the current aggregated datasets can be divided into six categories of four task types, as shown in Table 1. The functional programming language can be conducted to a new category in terms of trends, and the proposed research has mentioned this drift.

In particular, as a related field of code generation, semantic parsing aims to convert natural language utterances into logical forms with formal language. From our perspective, based on the complexity and abstraction of the formal language, we manually divide it into the simple logical actions and logical language. The simple logical actions belong to the most straightforward semantic parsing tasks. Some datasets possess a set of properties and actions that can be triggered by a pre-designed process, while some datasets attempt to describe the process control information (such as IFTTT) from a zones perspective. On the other hand, the logical language related datasets consist of natural language queries paired with a logical representation of their denotations. It has always been the most challenging branch task in the process of the semantic parsing field. There are various logical languages shown in datasets. The regular expression usually defines a pattern that verifies the feature of strings and has been widely used in various programming languages. As a more complex logical language initially employed to formal systems, lambda calculus applies a function to an argument and forms functions by abstraction [78]. Prolog can be converted to equivalent statements in the lambda calculus, where some of the datasets are annotated with Prolog-style semantics too. Further, lambda dependency-based compositional semantics (λ DCS) was proposed in 2013; it is an alternate formal language that can be notationally simpler than lambda calculus [79]. Many semantic parsing tasks have been based on λ DCS in recent years.

Domain-Specific Language (DSL) is very popular in the industry; it is a language designed for specific fields. The diversity between natural language and programs, which refers to the ambiguity of natural language, corresponds to the strictness of the program. Therefore, DSLG acts as a compromise to cope with this challenge, and the LISP-inspired DSL was designed for code generation instead of an existing programming language. It acts as a mediator to bridge the differences between natural language and source code. In addition, general-purpose DSLs like SQL and Bash are limited to the context of a specific platform or application and have been maturely applied in various development environments. Hence, the end-to end generation of DSL is becoming more accessible for recent SCG tasks.

The open-sourcing of the datasets promotes the automatic generation of source code snippets, especially the Trading Card Games (TCGs) like MTG and HS. TCGs map semi-structured information, including textual description and categorical numerical attributes, to general-purpose programming languages like Java and Python. Some new datasets focus on code snippet generation which is constrained by the programming framework and cross-domain features. These burgeoning datasets enrich code snippet generation in real scenarios instead of being limited to one mode. Moreover, some datasets are collected for program synthesis tasks concentrating on code generation under the programmatic contexts (CONALA) or the essence of API usage (AML) in general-purpose languages.

Appendix A shows the details of all the summed-up datasets. These datasets employ the input in natural language or other semi-structured methods and are subsequently converted into various languages. Some datasets contain additional contextual information that constrains the generated source code, such as the generated SQL having to be compatible with the database schema, and the addition of context information would impact the entire model design.

## 5. Insights Gained from NLSCG Research Backlog

**Figure 6:**
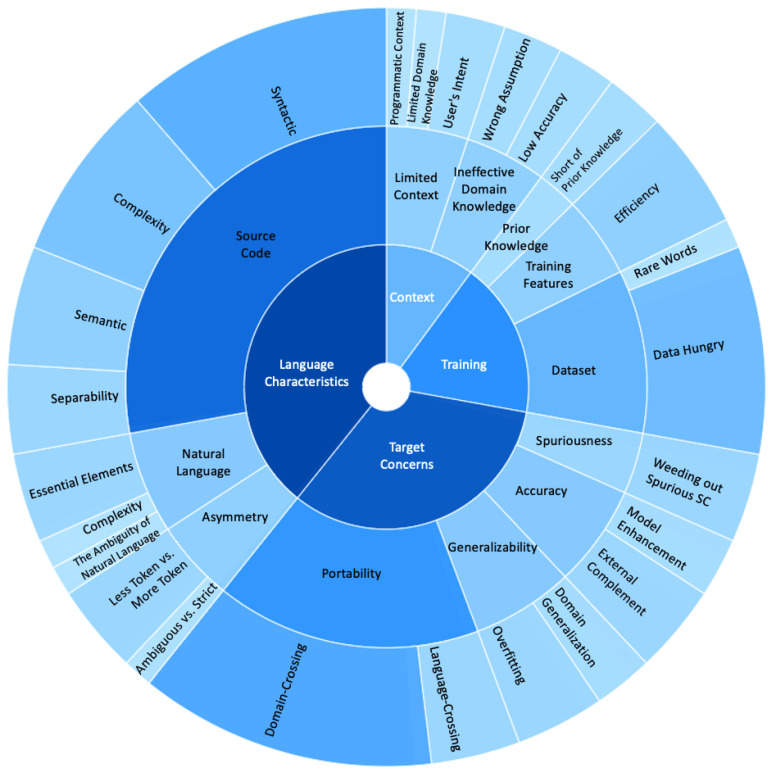
NLSCG Backlog Items.

With the continuous improvement of deep learning and NLP technologies, researchers have attempted to model NLSCG from new perspectives. Researchers tend to stand on the shoulders of giants and get used to enhancing models based on the previous classic approaches. Exploring their initial research concerns is critical, and helps us follow up with improving the direction of this field. Therefore, we extract the research concerns from the selected studies and abstract them into the NLSCG research backlog, expecting to bring a more glittering view to future research. As shown in Figure 6, four disciplines in the treemap specify the critical research tasks for attempts and possible improvement directions, while the three tiers indicate different levels of details. The amount of studies related to a given backlog item determines the corresponding area’s shade and size. We accumulate the research backlog in terms of resource, environment, boundary, enabler, utilities, etc., from the selected studies collected by the snowballing process. From the perspective of inherited research focuses and adaptive challenges, we summarize a taxonomy based on these research backlog items, which categorize all backlog items into four significant disciplines. (1) Language characteristics focuses on meeting challenges created by the inherent characteristics of natural language (NL) and source code (SC). (2) Target concerns emphasize the external features that are encountered during the generation process and that are able to be satisfied under a specific context or scenario. (3) Training refers to the difficulties confronted throughout the training process. (4) Context concentrates on processing the contextual information that exists in the input, output, and the entire generation problem. Despite this, “taxonomy” shows its immaturity and fragility; the scattered research backlog items gather together to present an overall comprehensible research treemap. Finally, we gain insights from the backlog of NLSCG research and elaborate the details in conjunction with specific studies.

### 5.1. Language Characteristics

Language is responsible for information transmission, and the mapping from NL to SC is one of the manifestations of abstract information transformation. NL and programming language possess various native characteristics that affect the model construction, and many targeted efforts have been put together to address such challenges. This subsection describes the insights gained from language characteristic items according to the input and output language category, which can be refined into the basic appearance, contributory factors, expected impacts, attempted solutions, etc.

When modeling the SC end, distinct characteristics, including syntactic and semantic, and latent characteristics, including complexity and separability, accumulate to this part of backlog items. Syntactic dictates ensure well-formedness, type-sensitiveness, and executability, and pay close attention to semantic equivalence, program aliasing, and semantic consistency between the two ends in a semantic aspect. The complexity of the inherent structure in the SC end brings difficulties to sequential modeling. Obstacles include long-distance dependency problems, long decoding paths, the insufficient separation between high-level patterns and low-level implementations, etc. It is necessary to make trade-offs between tracking complete details and training efficiency. Research tasks on the NL end present as almost identical to the regular NLP field, which can be summarized from essential elements, complexity, and ambiguity. The adaption to the NLSCG tasks has put new requirements on the language ambiguity, owing to the asymmetries between the NL and SC ends. These asymmetries are not only reflected to a strict extent, but also in the number of tokens between the two ends.

#### 5.1.1. Source Code

Syntactic. The generated SC must strictly satisfy a rich set of syntactic constraints, which can be easily extended to three aspects: Well-formedness, type-sensitiveness, and executability. To be specific, well-formedness can readily be kept by representing the SC as abstract syntax trees [4], while type-sensitiveness needs to guarantee the generated code is well typed. SC is often semantically brittle, and slight changes may drastically change the meaning of code [80], which may affect the executability. Besides, applying grammar-based decoding to general programming languages such as SQL is challenging as the AST may not sufficiently constrain its target [12]. Researchers devote themselves to tackle these fundamental problems through many different approaches, like formulating production rules [13], abstract syntax networks [4], program sketches [14], SQL template [15], targeted probability maximization [16], execution guidance [17], etc.

Complexity. The inherent structural information of SC induces it tricky-to-generate complex code correctly through the baseline approach. Take the case of RNN, which is considered as the primary fundamental network of the baseline approach. With RNN and its variants, memorizing large and complex structures is arduous [18], and capturing long sequences extracted from AST is inappropriate for RNN [19]. Besides, the long-distance dependency problem, occurring between code elements where their positions are far apart in space [20], exacerbates the unsuitability of RNN-based modeling. What is more, the process of converting SC into a flattened sequence through AST can easily lead to long decoding paths, even for short snippets of SC [21]. Some over-detailed expansions will not provide fancy information for constructing neural decoding models; instead, this will bring in interference and result in a meaningless waste of training resources. Complexity also affects the DSLG tasks, especially the generation of nested SQL queries [22], and we can simplify the complexity of the generator and improve performance by ignoring the order of fields and condition attributes [23].

Semantic. Prevalent challenges exist across diverse SC categories in the aspect of semantic and syntactic elements. Meanwhile, slight differences appear among these elements in complexity and significance. In terms of semantics, Xu [24] finds that different SQL queries may be equivalent to each other due to the commutativity and associativity, which lead to the futility of the sequence in certain SQL positions, like tokens in attributes in SELECT and WHERE clauses. However, the order of these constraints would affect the performance of a sequence-to-sequence-style model, which is considered as the baseline model in the SCG tasks. In addition, many different programs can frequently satisfy one given the NL specification, which suggests a phenomenon known as “program aliasing” [16,25], and this type of limitation pervades all SC categories.

Separability. Program synthesis is perceived as the most challenging of the intelligent NLSCG tasks due to the insufficient separation between high-level patterns and low-level implementation details [7]. The low-level features, such as variable names and intermediate results, would impede the learning process. While the high-level patterns are challenging to learn from datasets, a combinatorial, syntax-guided program synthesizer can easily accomplish this task [81]. Therefore, researchers [14,26] have attempted to get the decomposition disentangled high-level features from low-level features, and enable decoders to model at different levels of granularity.

#### 5.1.2. Natural Language

Essential Elements. On account of the two essential elements of natural language, vocabulary and syntax, tentative efforts have been made to get improvements. Some studies merely extract word order features depending on SeqLSTM while neglecting useful syntactic information [27]. To address the problem, authors [28] tried to employ syntactic trees (like CCG and CFG) instead of flat sequence on the input end to thoroughly capture NL information based on dependency parsing and constituency parsing . Meanwhile, it is noticeable that the characteristics of RNN structure make it difficult to learn proper representation from the diverse vocabulary and syntax [29].

Complexity. In particular scenarios, the input question composed of sub-questions might be too long to understand. Understanding each sub-question could contribute to the semantic parsing of the original complex question [30]. The decomposition of complex questions can be considered as the predecessor task of NLSCG to alleviate the difficulties of understanding. Rule-based methods have been proposed [82], but experts and massive design efforts have to be devoted to the task. A neural question decomposer has been tentatively proposed to identify sub-questions more accurately [30].

Ambiguity. Another problem that blocks the research process is language ambiguity [31]. After a long-term NL evolution, people tend to express their thoughts relatively concisely, such as with fewer words and shorter phrases. More importantly, ambiguous language can be accurately understood only on the premise that the whole context is comprehended. The linguistic ambiguity problems may affect the meaning of the syntactic elements [83], which aggravates the difficulty of generating the corresponding code.

#### 5.1.3. Asymmetries between Natural Language and Source Code

Encoder–decoder architecture is widely applied to intelligent NLSCG and its similar tasks. The encoder takes NL as the input sequence, converts it into specific representations, and then transforms it into the desired code structures via the decoder. This section attempts to summarize the research backlog affected by the asymmetries between encoder and decoder.

Less Token vs. More Token. Many studies find that apparent asymmetries in the number of tokens between input and output end give rise to beset difficulties in building the generative models [18,19,25]. These asymmetries can easily attribute to the larger number of tokens in the source code end. To ensure source code can be modeled without missing rich structure information, converting source code into tokens depending on a specific traversal approach from AST is prevalent. Extracting tokens on account of the tree-based approach can easily lead to rapid quantity growth compared to the original source code, which exacerbates the appearance of asymmetry. CNN-based models [19], retrieval-based generation [18], and pre-training distributions of SC and NL [25] are proposed to address this backlog item.

Ambiguous vs. Strict. As an essential attribute of NL, ambiguity brings difficulties in describing the exact requirements clearly [9]. In contrast, the source code seems to be sophisticated equipment with precision and unequivocal representation. A slight difference would impact on syntax, executability, and even semantics of source code. Therefore, extra efforts must be devoted to coping with the transformation from ambiguous language to strict language. Research [9] tackled this problem by attaching input and output examples to synthesize programs.

### 5.2. Target Concerns

Concerns running through the entire modeling process are worthy of consideration, and many studies attempt to concentrate on it to get potential improvements. In this section, we summarize the four chief features that researchers devote more attention to. We inspect the portability and generalizability from a global perspective, and the accuracy and spuriousness focus on evaluating the generation task itself, where we discuss their derivation, usage scenario, and tentative solutions. The concerns expressed by these models can be applied to evaluate the external model learning ability to a certain extent. By studying the current limitations of these concerns, favorable directions to improve the model became apparent.

For achieving better performance, researchers restrict the model to a limited domain, resulting in domain-crossing problems. The new release of cross-domain datasets and attempts at learning methods have greatly alleviated this difficulty. Since the semantic equivalence between the two languages cannot be calculated, it is almost impossible to construct a cross-language generative model under the existing technical conditions. This backlog item still has a long way to grow. In terms of generalization ability, overfitting is a common problem, and recursive generative architecture and reinforcement learning methods have been presented to tackle this problem. External complement and model enhancement perspectives can improve the accuracy of the model. Dataset augmentation, search space optimization, dialog-based generation, and two-stage generation are prevalent approaches in external complement. Researchers are improving the model accuracy by discovering potential limitations in architecture strategies and learning approaches. Lastly, to solve spurious programs, the auxiliary reward function of reinforcement learning and the mechanism to eliminate spurious programs during the training process are practical approaches.

#### 5.2.1. Portability

Domain crossing. Generating target code with high precision across domains has become the main bottleneck of NLSCG tasks tightly coupled with domain knowledge. As elaborated in Section 5.4.2, domain knowledge utilization shows progressive features, (1) investigating whether to utilize domain knowledge in the budding phase; (2) discussing how to utilize domain knowledge in the rising phase efficiently; and (3) addressing how to utilize domain knowledge sufficiently under the premise of crossing domains in the perfection phase. The inherent ambiguity exhibited by NL restricts the NLSCG task to specific application scenarios [32]. Depending on manually designed features, many previous works build models with a domain-specific specialty [33], and these models learn to match semantic parsing results rather than truly learn to understand the meanings of inputs [34], making it challenging to adapt to new domains. Building the model targeted on one specific domain (i.e., database, table) [35] or binding interactive guidance from users has become the primary approach that existing technologies try in order to achieve high accuracy. Hence, existing techniques for NL-based synthesizing of the corresponding code , SQL in particular, are not database agnostic (i.e., do not require database-specific training or customization) [36]. One reason is that the training datasets applied in the NLSCG tasks based on supervised learning are concentrated on the particular domain while ignoring data previously collected for other domains. The popular Overnight [84] dataset containing eight domains has greatly alleviated this problem. In addition to mainstream supervised learning, some general cross-domain learning categories have been introduced to NLSCG, including meta-learning [37] and transfer learning [38], and have achieved confident performance.

Language crossing. NLSCG tasks embrace various code types constrained by relatively unique grammatical rules. To achieve the general-purpose generation and ensure the syntactic well-formedness of the generated code, it is necessary to design a parser that explicitly reflects the domain-dependent grammar of code embedded in the model [39]. Prior work proposes a model that performs the task from text descriptions to regular expressions, utilizing a domain-specific component that computes the semantic equivalence between two regular expressions [85]. However, the effectiveness of this approach is extremely dependent on the component mentioned above, leading to trouble with generalizing to other languages since the semantic equivalence calculations are not possible across other formal languages [40]. Therefore, addressing this issue with general-purpose grammar models has become a considerable exploration direction.

#### 5.2.2. Generalizability

Overfitting. Overfitting remembers superfluous minor features of the training data and reduces the generalization ability. A model with good generalization must be able to perform exceptionally well outside the training set. In terms of the NL end, due to the particularity of real-world NLSCG tasks, the large and varied vocabulary with many rare words naturally intensifies overfitting [41]. When it comes to the SC end, the more extraordinary complexity generation scenarios, like the multi-schema problem in SQL, would aggravate the occurrence of overfitting [42]. Recent work [10] has discovered that incorporating the notion of recursion into neural program synthesis architecture or leveraging reinforcement learning instead of supervised techniques could significantly improve model generalizability.

Domain Generalization. Solving the problems of limited training samples and data distribution conversion can improve the domain generalization problem to a certain extent. As described in Section 5.4.1, domain crossing can evaluate the portability of a model, which means training based on one domain and then smoothly applied to another one without any additional training data. However, it is challenging to achieve this goal as, in the training process, not all the target domain KB constants (relationships and entities) can be observed at the training stage [15], and emerging domains will break the pre-adapted models. Furthermore, mainstream complex SQL query generation methods often take SQL templates or slots as the intermediate absorber and impair the ability to generate code of unseen templates [43].

#### 5.2.3. Accuracy

External Complement. Improvements based on the model itself are prone to touch the ceiling. From an engineering thinking perspective, complementing specific components externally, the model can more effectively improve the performance of the generated tasks. Inspired by successful application in machine translation, a retrieval-based approach has been introduced into neural code generation [18]. For instance, studies [6,44] gain an insight that the parser still maintains high recall when incorporating the n-best predictions into the gold-standard generated code most of the time. Thus, by reranking the n-best list of candidate code snippets, they investigate potential actions to improve the precision.

Model Enhancement. Popular external complements, such as training set enrichment [45], search space optimization [6,18,46], purposeful supplementation of context information [32], two-stage generation [7,14,36], etc., are prevalent approaches to improving the accuracy of NLSCG tasks. However, some researchers are dedicated to focusing on a pure end-to-end approach. They learn experiences from historical success models, discovering latent limitations from the perspectives of model architecture strategy and learning approach, and improve precision within an accomplishable range. Research [8] has proposed a bare neural model, without any search, additional postprocessing, or other forms of auxiliary means, and still achieved state-of-the-art test-set accuracy. Instead of designing multiple decoders from different language components or adding extra controllers for expansion of production rules, authors [47] have proposed a fine-grain control over the decoding process while retaining the simplicity of the sequence structure.

#### 5.2.4. Spuriousness

Weeding out Spurious SC. NLSCG sometimes encountered spurious programs, which are prevalent in any general-purpose programming language. Spurious programs denote that the incorrect utterances accidentally produce correct results yet do not reflect the actual semantics. Research [48,49,50] in selected studies explored this topic. Spurious programs would provide wrong signals for the generation model, developing in an increasingly biased direction. This can be alleviated by using the auxiliary reward function derived from reinforcement learning [48] or designing a mechanism to eliminate spurious programs during training [50].

### 5.3. Training

Sometimes the failure of training neural networks occurs in silence; Karpathy [86] has proposed a recipe to achieve better performance for training neural networks. Compared with adopting inertial and experience-based tricks with a kind of elusiveness, researchers are trying to find the breakthrough from visible limitations, including training dataset and training characteristics, to get improvements. In this subsection, we summarize the research backlog items related to training that researchers discover in the NLSCG field to inspire potential enhancements.

Data-hungry and rare word problems are widespread in machine learning training tasks. The former can be alleviated via semi/unsupervised learning, data augmentation, and releasing new datasets to a certain degree. In dealing with rare words problem, the only thing to pay attention to is how to accurately embrace the successful experience applied in other fields to the distinctive SC end. Capturing all tiny details on the SC end would inevitably lead to computational challenges, and researchers should make trade-offs between complicated deep learning models and training efficiency. In addition, injecting prior knowledge would improve model performance, and successful attempts are based on the data recombination process.

#### 5.3.1. Dataset

Data Hungry. The dataset is one of the essential factors determining the upper bound of the model performance to some extent. The abundance of high-quality labeled data is critical for effectively training supervised models [31,51]. However, manually annotating NL utterances with their corresponding SC is expensive, cumbersome, and time consuming [10,38,52,53]. While emerging datasets have sprung up, due to the limitations in quantity, quality, and domain-crossing, the limited availability of labeled data is still becoming the primary bottleneck for data-driven supervised models [49].

Rare Words. It is challenging to learn representations of rare words with a neural language model. The entire word sequence containing rare words would be underestimated due to the influence of rare words on the context of neighboring words [87]. Words that are rare in the dataset are unable to learn good enough parameters [88]. The hybrid method combining significant example retrieval and neural models has been proven to be successful in processing rare words [89]. Hayati et al. [18] deal with rare words through constructing n-gram over subtrees to exploit the code structural similarity.

#### 5.3.2. Training Features

Efficiency. Modeling source code with large formal grammars can easily lead to long decoding paths, even for short code snippets [21]. The training of deep neural networks with complex topologies is difficult, time consuming, and error-prone [54]. Researchers should find a way to make a trade-off between complicated deep learning models and training efficiency. Zhong et al. [55] discovered that the output space of the softmax in the Seq2Seq model would be unnecessarily large if the generated sequence cannot be restricted to a particular list. Computational challenges occur concerning supervised learning, as well as concerning semi-supervised learning. A semi-supervised autoencoder tends to convert the transformation object from (x→y) to (y→x→y) by considering the input sequence as a latent variable, where x is the input sequence, and y represents the output sequence. However, modeling the latent variable as a series of discrete symbols drawn from multinomial distributions will bring serious computational challenges, as it requires marginalizing over the space of latent sequences $\sum_{x}^{*}$ [51].

#### 5.3.3. Prior Knowledge

Lack of Prior Knowledge. Recently, some NLSCG tasks tried to build more flexible models and generate the corresponding code via learning more general grammars and features while trimming lots of task-specific prior knowledge. Compared with standard semantic parsers, this modeling flexibility is attributed to the common application of minimal feature engineering represented by RNNs. Such knowledge plays a critical role in understanding modeling limitations, and the parser would get better performance under the constraints of task-specific prior knowledge [56]. Jia and Liang [57] have proposed a data recombination framework which induces a generative model from the training data and samples from it to generate new training examples with available prior knowledge.

### 5.4. Context

The context indicates information about the circumstances in which something or someone is located, which can influence and assist users in understanding the original intention to a certain extent. In the entire process of NLSCG, three components would be affected by the context, including the input end, the output end, and the relatively special domain knowledge across the whole generation problem (especially for instances in DSLG and SP). The lack and insufficient application of such information impel us to discover these research backlog items.

However, rare existing works make sufficient use of the context information, which accumulates the backlog items of ineffective domain knowledge. The inefficiency is mainly reflected in the low accuracy caused by the lack of accurate knowledge embedding and the preconceived wrong assumptions before model design. Besides, the limited context indicates the restricted context from the user’s intents and programmatic and generation problem perspectives. The present items mainly lie in the narrow scope of the context, insufficient utilization manner, and lack of reasonable solutions, etc.

#### 5.4.1. Ineffective Domain Knowledge

Low Accuracy. The input NL query often contains keywords that can be specific to the underlying database. Shreds of evidence show that those words are crucial to many downstream actions , including inferring column names and condition values in the SQL query [58]. Some particular tasks of NLSCG constrained by additional domain knowledge to generate proper SC present significant differences in taking advantage of domain knowledge. Research [59] notices that some studies do not utilize domain knowledge; specifically, those missing or lacking accurate embeddings exist between the database schema entity and the input query.

Wrong Assumption. Researchers [43,58] find many models assume that users have an exceptionally familiar sense of the domain knowledge, such as knowing about the exact column names and string entries in that table. They copy the question words from the input query directly via a soft-alignment mechanism. However, the mismatch between question words and column names (or cells) exacerbates the incorrectness and non-executability of the generated results. Under these circumstances, even if the embeddings of domain knowledge reach perfection , the performance of downstream tasks could not meet the expectations, and it seems to be counterproductive occasionally.

#### 5.4.2. Limited Context

User’s Intent. Acquiring users’ real intentions follows the principle of gradual improvement. However, the reality is that existing works tend to assume that the corresponding source code can be generated end to end from one single NL description [60]. Therefore, many users’ fundamental details are omitted, yet the generator does not have the capabilities to figure out the user’s real intentions via their initial description.

Programmatic Context. The mainstream generating approaches of NLSCG have restricted the relevant code to a limited language and code environment. In most cases, the generated code focuses on more straightforward scenarios and context, so that very few of them take into account programmatic context. For example, the fixed code templates generate only parts of a method with a predefined structure, such as IFTTT and custom actions; a fixed context generates the body of the same method within a single fixed class, such as code snippets; or no context at all generates code tokens from the flat SC sequences separately, such as SQL slots [61]. All those mentioned above are still a long way from the executable program. While these fragments have motivated NLSCG solutions in model design and architecture selection, the generation scenario with the programmatic context is more complicated and presents its practicality.

Simple Context. In NLSCG tasks, mapping NL utterance to the target SC under the context of special domain knowledge, such as generating SQL constrained by database schema, semantic parsing associated with its own knowledge base, is a particular instance. The number of domains, schemas, and tables, along with users’ intentions, can determine the difficulty level of SQL generation tasks . Many datasets about one domain contain only one database schema for that domain; more seriously, some schema only contain one table [42]. Consequently, many existing works are often formed into simple and crude applications, as complex scenarios like multi-table cascade queries by foreign key can never happen concerning these works.

## 6. Perspectives on NLSCG Latest Technology Landscape

This section summarizes the foremost important part of the NLSCG models through an intensive investigation of the selected studies. The generative model can be regarded as a particular data processing problem, with which it is easy to depict the entire generation process clearly by utilizing a pipeline-based approach. Based on the two main processing pipelines of NLSCG, namely the NL end (description of requirements and intents) and the SC end (executable source code in a specific context); regarding research backlog as guidelines, we decompose raw information from selected studies, and convert them into essential components, and then build interactions according to the data processing chain and the conventional deep learning model pipeline. We propose a latest NLSCG technology landscape illustrated with a metro map metaphor, to explain the NLSCG utilities as vividly as possible. On this basis, the leading technical tactics adopted by each component are elaborated gradually to stimulate more possibilities for model construction.

**Figure 7:**
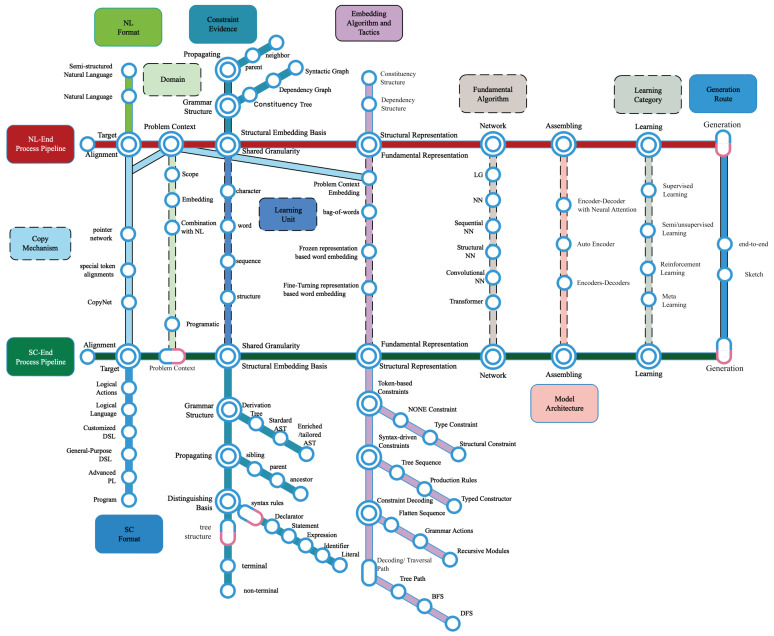
NLSCG Technology Landscape.

Regarding NLSCG tasks as data processing problems, we use the pipeline-based diagram to produce a thorough elaboration of the current utilities shown in Figure 7. Like two ends of a scale, we could go through two main routes covering the generation process from NL to SC when traveling along with pipelines in the landscape diagram. Besides, another 11 routes run through the main routes directly or indirectly with the identification of corresponding color and guideboard. During the crossroad, important components are highlighted in the form of different types of shapes. The critical technical points are displayed in the diagram in the form of a circle point. The solid path indicates that information can be directly transmitted between the two main lines, while the dashed path cannot. Table 2 describes the significant routes and intersections deriving from the landscape. Here we consider them as the actuating routes and enabling factors that motivate the further improvements of the NLSCG field. The representative studies which indeed describe the corresponding enabling factors are also exhibited in this table.

NLSCG maps natural language (NL) utterances into an executable representation. As mentioned in Section 4.3, these executable representations refer to logical actions, logical language, customized DSL, general-purpose DSL, advanced programming language, and program. There are some scenarios where the NLSCG process depends on a specific programmatic context [61]. The input representation is typically executed against a problem context, such as a knowledge base [62] or database [58], to generate the desired output. For the problem context that plays an essential role in semantic analysis and domain-specific language generation tasks, the context scope, the context-embedding approaches, and the manners of combining context with the NL end are worthy of attention.

Different from the ambiguity of natural language, the source code is constrained by an underlying syntax or grammar. Such constraints can usually be converted into an intermediate structure with AST as the typical grammar criterion. The complexity of the intermediate tree structure brings difficulties in leveraging information with rich structures, which attracts most researchers’ attention. The prerequisite for taking full advantage of such information is to clarify the structural embedding basis before gaining representations, which is different from the conventional word or character level granularity of the NL. The inherent structural information in the SC constrained by the syntax rules has shown its utilization characteristics of diversity; different state propagation directions, grammar conversion criterion, distinguishing basis, and the random combinations between them have a profound impact on the design of subsequent embedding algorithms. Considering the complete embedding for both ends, researchers take sequence or structure as the modeling object to construct the network. Researchers also make trade-offs between the levels of details and the final network complexity in the structure-embedding process to ensure the training efficiency of the model.

After confirming the granularity of the learning starting point, the relevant NL should be encoded into the representations that the computer can recognize, with the expectation of not losing information during the encoding process. At this stage, comparing NL and SC, current explorations present a relatively different situation and tendency. Overall, various embedding algorithms are applied to the NL end, while few researches have focused merely on the bare source code encoding. The majority of the NL end and minority of the SC end are regarded as flat sequences, adopting representation learning algorithms commonly applied in the NLP field. In contrast, both ends attempt different capture strategies for extracting information via the structure modeling object. By employing the syntactic analysis thinking, the NL end directly builds tree-based neural networks on the intermediate syntactic structure. The representation of structural information from the SC end shows its own particularity, with the granularity and structural embedding basis as the cornerstones. These foundations serve as inspirations to design constraint strategy and decoding process, and gradually develop into mature approaches. Current studies have made efforts in two types of constraints, namely token-based constraints and syntax-driven constraints; combining specific constraints with the appropriate decoding process would unleash huge creative potentials. These constraints and decoding processes establish a benchmark for accurately tracking structural information. Further, various methods are branched on this mainline to perform subsequent modeling and deriving to different factions.

To ensure the syntactic correctness and semantic accuracy of the generated SC, the constraint decoding attaches critical structural constraints to the decoding inference stage. Afterward, a distribution is produced over these SC by modeling and ultimately screening high scoring results after the searching procedure under this model. Similar to familiar intelligent generation tasks, the fundamental algorithms adopted by NLSCG are based on popular deep learning models and their variants. These actualities hold for both NL and SC end modeling. Multiple types of comprehensive networks, including sequential NN (RNN, LSTM, etc.), convolutional NN (CNN), and structural NN (tree NN and graph NN), are attempted in this field, and the emerging modeling algorithms like Transformer are applied to NLSCG and have gradually shown their superiority.

In most cases, the complexities of NLSCG tasks result in the infeasibility of generation relying on one single neural network. Therefore, the encoder–decoder framework with neural attention has become the baseline architecture of NLSCG, and the assembly of various models on this basis is also becoming increasingly mainstream. The attention mechanism was initially introduced to align the information between NL end and SC end, and it is frequently employed in the splicing and integration process of the network.

The learning algorithm is employed to update the model’s parameters on account of the training dataset. Supervised learning still occupies the mainstream category with its more accessible training and higher accuracy. Semi/unsupervised learning has also been introduced as the auxiliary method to solve the data-hungry problem. Reinforcement learning has also been introduced to enhance performance and generalization. In addition, meta-learning is constantly being introduced into this area to deal with cross-domain problems.

Hybrid methods have proven to be effective in NLSCG tasks. Hence, we could transfer between different routes from the starting, terminal, and intermediate points in the landscape to inspire potential improvements of NLSCG utilities. The end-to-end generation strategy sustained by technologies and components linked by the pipeline still occupies the mainstream position of the current generation task’s technology stack. However, many studies shed a different light on external complementary methods, with the hope of making the next leap in performance and domain-crossing. Genres based on search concepts, including combining retrieval methods into neural code generation models [18,46], reranking a list of potential candidates [6], etc., are relatively feasible. Besides, data augmentation [45,63] processing based on training datasets , as well as the two-stage [52] and sketch [24]-based generation tactics, are also worthy of consideration.

## 7. Conclusions and Future Directions

SCG has been studied for a long time, and the NLSCG shows its popularity with the increasing adaptation maturity of deep learning techniques. This survey provides insights for NLSCG from laborious and deliberate exploration of 66 relevant studies collected by the well-designed snowballing process. We extract preliminary information from the selected studies, review the overall development trend in recent years, summarize the frequently used dataset, and refine the types of NLSCG research tasks, consequently promoting the understanding of the NLSCG problem. Then the research backlog is filled by considering critical motivations and priorities under the guidance of the research journey map. We extract a taxonomy based on the research backlog items through a circular treemap regarding the inherited research focuses and adaptive challenges. Then we summarize the insights gained from the NLSCG research backlog and elaborate the details. Finally, based on the two main processing pipelines of NLSCG, we propose a technology landscape of NLSCG to explain the NLSCG utilities as vividly as possible. The technology landscape depicts the critical fundamental components, correlations between components, and leading technical tactics, stimulating more possibilities for model construction.

We agree that the following issues need to be addressed in future work. First of all, there is no precise horizontal comparison between the selected studies. The reasons are as follows: (1) There are significant differences in various types of generation tasks, which hinder the comparison; (2) most selected studies use different datasets and evaluation benchmarks, which increase the difficulty of comparison. Secondly, this article only introduces main components in the NLSCG landscape due to space limitations, while ignoring important details about each technical solution, which need to be elaborated in future research.

## Figures and Tables

**Table 1:** Output-oriented Dataset Classification.

Classification	Generation Object	Dataset Name	Identifier
Simple Logical Actions	IFTTT	IFTTT 2015	1.1
		IFTTT 2016	1.2
	Custom Actions	SAIL	1.3
		SCENE	1.4
		ALCHEMY	1.5
		TANGRAMS	1.6
		Karel	1.7
Logical Language	Regular Expression	NL-RX	2.1
	Prolog	JOBS	2.2
	Lambda Calculus	GEO	2.3
		ATIS	2.4
		NLMAPS	2.5
		SPADES	2.6
		GeoGrAnno	2.7
	Lambda DCS	WEBQUESTIONS	2.8
		OverNight	2.9
		WikiTableQuestions	2.10
		WEBQUESTIONSSP	2.11
Customized Domain Specific Language	LISP-inspired DSL	ALGOLISP	3.1
General-Purpose Domain Specific Language	Graph Query	GRAPHQUESTIONS	4.1
		ComplexWebQuestions	4.2
	SQL	GEO	4.3
		ATIS	4.4
		WikiSQL	4.5
		SENLIDB	4.6
		Scholar	4.7
		Textbook	4.8
		Advising	4.9
		Spider	4.10
		MIMICSQL	4.11
	Bash	NL-Bash	4.12
Advanced Programming Language	Python	DJANGO	5.1
		HS	5.2
	Java	MTG	5.3
		ACBJ	5.4
Program	API Usage	AML	6.1
	Programmatic Context	CONALA	6.2
		CONCODE	6.3

**Table 2:** Significant Enabling Factors in the Landscape.

Actuating Routes	Enabling Factors	Description	Representative Studies
Copy Mechanism	Alignment	Outline the most classic and widespread strategies that align the NL end and SC end.	S2,S3,S6,etc.
Domain	Problem Context	Focus on the critical contextual information and how it assimilates into the generative model.	S7,S34,S35,S45, S56,S63,S65,etc.
Learning Unit	Shared Granularity	Sketch the granularity of the participants’ vectorization unit in the encoding stage (word or character) and modeling object in the network construction stage (sequence or structure).	S1,S22,etc.
Constraint Evidence	Structural Embedding Basis	Discuss the basis for achieving structural embedding from different views.	S2,S3,S14,S36, S50,S58,S64,etc.
Constraint Evidence	Grammar Structure	Describe the extraction criteria for getting intermediate structure.	S36,S58,S64,etc.
Constraint Evidence	Propagation	Discuss the additional state propagation between nodes at the different relative position in the grammar structure.	S6,S9,S21,etc.
Constraint Evidence	Distinguishing Basis	Indicate the distinguishing basis of different nodes in the grammar structure in the subsequent modeling process.	S2,S3,etc.
Embedding Algorithm And Tactics	Fundamental Representation	Investigate the conventional embedding algorithms and tactics based on the original sequence input.	S1,S8,S15,S30,etc.
Embedding Algorithm And Tactics	Structure Representation	Explore the common embedding algorithms and tactics based on the intermediate structure.	S11,S14,S15,S50, S66,etc.
Embedding Algorithm And Tactics	Token-based Representation	Summarize the embedding strategies that consider tokens as the decoding object and hold the extended constraint during the decoding process.	S5,S7,S16,S20, S26,S34,etc.
Embedding Algorithm And Tactics	Syntax-driven Constraints	Summarize the embedding strategies that utilize intermediate structure as the springboard, granularity and syntactic constraints as the foundation and standard, and they work together to extract the structure information.	S2,S3,S11,S15, S25,S44,S45,etc.
Embedding Algorithm And Tactics	Constraint Decoding	Summarize on the strategy for attaching critical constraints to the decoding inference stage to ensure the syntactic correctness and semantic accuracy of the generated result.	S2,S3,S8,S10, S11,S15,etc.
Fundamental Algorithm	Network	Introduce the widely adopted networks and variants and the approaches to integrating the learning unit, constraint evidence, and embedding algorithm and tactics.	S1,S7,S11,S14, S22,S23,S52,etc.
Model Architecture	Assembling	Outline the assembling paradigms that assemble multiple encoders or decoders composed of fundamental networks.	S8,S11,S15,S23, S51,S63,etc.
Learning Category	Learning	Summarize the learning categories that NLSCG attempts and their adaptive scenario and superiorities.	S8,S15,S16,S24, S28,S30,S55,etc.
Generation Route	Generation	Discuss the generation manner in the way of end-to end or via an intermediate stage.	S6,S34,S35,S41,etc.

## Data Availability

Selected Studies List Ref: https://github.com/Ada12/NLSCG_Research/blob/main/selected-studies-list.md, accessed on 20 August 2021.

## Abbreviations

The following abbreviations are used in this manuscript:

NLSCG	Natural Language based Source Code Generation
SCG	Source Code Generation
NL	Natural Language
SC	Source Code
EMA	Enriched Models based Approach
DSLA	Domain-Specific Language based Approach
FLA	Formal Language based Approach
GMA	Graphic Modeling based Approach
NRA	Nature Requirement based Approach
SP	Semantic Parsing
DSLG	Domain Specific Language Generation
CSG	Code Snippet Generation
PS	Program Synthesis
TCGs	Trading Card Games

## Appendix A. Dataset List

**Table A1:** Datasets.

ID	Name	Output-End Form	Scale	Year	Related Study
1	JOBS	Prolog-style Queries	640	1996	3,11,50
2	GEO	Lambda Calculus / SQL	880	1996	41,50,54,57,60,65
3	SAIL	Custom Actions	3236	2006	28
4	ATIS	Lambda Calculus / SQL	5871	2010	41,46,50,57,58,60,61
5	WEBQUESTIONS	Lambda DCS	5810	2013	29,37,44,54
6	IFTTT 2015	If-This-Then-That Recipes	86,960	2015	2,5,13,25
7	DJANGO	Python	18,805	2015	1,2,8,10,12,17,65
8	OverNight	Lambda DCS	13,682	2015	38,39,49,60,63
9	MTG	Java	13,297	2016	1
10	HS	Python	665	2016	18,22,23
11	WikiTableQuestions	Lambda DCS	222,033	2016	4,59
12	IFTTT 2016	If-This-Then-That Recipes	291,285	2016	25,48
13	NLMAPS	Lambda Calculus	2380	2016	28
14	SCENE	Custom Actions	4403	2016	30
15	ALCHEMY	Custom Actions	4560	2016	30
16	TANGRAMS	Custom Actions	4989	2016	30
17	NL-RX	Regular Expression	10,000	2016	31
18	SPADES	Lambda Calculus	79,247	2016	37,54
19	GRAPHQUESTIONS	Graph Query	5166	2016	37,40,54
20	WikiSQL	SQL	80,654	2017	42,55,57,59,62
21	WEBQUESTIONSSP	lambda DCS	4737	2016	14
22	SENLIDB	SQL	24,890	2017	21
23	Scholar	SQL	817	2017	32
24	Textbook	SQl	108	2017	35
25	NL-Bash	Bash	5413	2017	40
26	AML	AML	150,000	2018	6
27	ALGOLISP	DSL inspired by LISP	100,000	2018	9,15
28	CONALA	Python	2879	2018	17
29	Advising	SQL	4385	2018	24
30	Karel	Customer Tree	10,000	2018	43
31	CONCODE	Java	100,000	2018	19,45,46
32	Spider	SQL	10,181	2018	18,47,51,56,58,66
33	ComplexWebQuestions	SPARQL	34,689	2018	52
34	GeoGrAnno	lambda calculus	824	2019	63
35	ACBJ	Java	1,074,963	2019	64
36	MIMICSQL	SQL	10,000	2020	53
